# Clinical Application Perspectives of Lung Cancers 3D Tumor Microenvironment Models for In Vitro Cultures

**DOI:** 10.3390/ijms23042261

**Published:** 2022-02-18

**Authors:** Irena Wieleba, Kamila Wojas-Krawczyk, Paweł Krawczyk, Janusz Milanowski

**Affiliations:** Pneumonology, Oncology and Allergology Department, Medical University of Lublin, 20-059 Lublin, Poland; kamilawojas@wp.pl (K.W.-K.); krapa@poczta.onet.pl (P.K.); janusz.milanowski@umlub.pl (J.M.)

**Keywords:** lung cancer, tumor microenvironment, organoids, spheroid, tumor-on-chip

## Abstract

Despite the enormous progress and development of modern therapies, lung cancer remains one of the most common causes of death among men and women. The key element in the development of new anti-cancer drugs is proper planning of the preclinical research phase. The most adequate basic research exemplary for cancer study are 3D tumor microenvironment in vitro models, which allow us to avoid the use of animal models and ensure replicable culture condition. However, the question tormenting the scientist is how to choose the best tool for tumor microenvironment research, especially for extremely heterogenous lung cancer cases. In the presented review we are focused to explain the key factors of lung cancer biology, its microenvironment, and clinical gaps related to different therapies. The review summarized the most important strategies for in vitro culture models mimicking the tumor–tumor microenvironmental interaction, as well as all advantages and disadvantages were depicted. This knowledge could facilitate the right decision to designate proper pre-clinical in vitro study, based on available analytical tools and technical capabilities, to obtain more reliable and personalized results for faster introduction them into the future clinical trials.

## 1. Introduction

Cancer is a leading disease in mortality worldwide. Tremendous progress in cancer diagnostic and therapies has been made in the past years. Tumorigenesis consists of three main stages: initiation, promotion, and progression. During initiation and promotion, normal cells change their phenotype, based on anchored genetical mutations, into a cancerous one. One may observe increasing metabolomic, intensive cell differentiation within changes in tumor stroma mechanics and increased cell mobility. In the progression stage interaction between tumor cells, stromal cells and immune cells provide to neovascularization and metastasis initiation [1,2]. During the epithelial–mesenchymal transformation (EMT) cancer cells develop a mechanism of immunosurveillance and drug resistance. For better analysis of this process in an in vitro model, it is required to use tumor microenvironment (TME) elements together with cancer cells. One significant technical aspect, which cannot be solved in classical 2D in vitro cell culturing, is tumor heterogenicity. Next, interaction between tumor and its stromal cells is investigated, as well as interaction between tumor and tumor infiltrated immune cells. Cell-to-cell interactions are regulated by specific molecular pathways. This type of interaction and their input on tumor progression cannot be clearly examined during cell culturing in monolayer [3,4,5]. The golden mean of 3D culturing technics is a precision imaging on tumor spatial organization ex vivo. The development process for new therapeutic or new diagnostic methods starts from basial research. The key point to gain more applicable preclinical studies is the selection of the most appropriate analytical tool, which will give us the possibility to applicate conducted data into clinical practice. The aim of this paper is to summarize the main directions in three-dimensional (3D) culturing technologies with the aim of solving challenges from lung cancer diagnosis and treatment. 

Lung cancer (LC) is a cancer type leading in morbidity and mortality rate worldwide. There are about 11 genetically differentiated and 2 main histopathological subtypes of lung cancer. Non-small cell lung cancer (NSCLC) refers to 85% of all lung cancer cases. Another 15% are classified as small-cell lung cancer (SCLC), which is more aggressive in the metastatic stage [6]. Therapeutical options for lung cancer treatment include surgery, stereotaxic radiotherapy, chemotherapy, targeted therapy, and immunotherapy. A major percentage of LC are diagnosed in the advanced disease stage. The main therapeutical strategy for non-operative patients in the IIIB–IV disease stages is a combination of chemotherapy and targeted or immunotherapy. For patients with driver mutations, such as EGFR (10% of NSCLC cases) and *BRAF* (0.5–4.9% of lung adenocarcinomas), and rearrangements in *ROS1* (1–2% of lung adenocarcinomas) and *ALK* (3–7% of lung adenocarcinomas) gene-targeted therapy can be used [6,7]. Nowadays, the main immune therapy strategy used for LC treatment is immune checkpoint inhibitors (ICIs). The introduction of ICI therapy into clinical practice gave a significant elongation in progression-free survival time and overall survival time, but the data are not highly satisfactory. High tumor mutational burden, microsatellite instability, and programmed death ligand 1 (PD-L1)-positive expression are used as predictor factors for ICI immune therapy and are still under investigation [8]. Nevertheless, in standard clinical practice, only PD-L1 expression on cancer cells with immunohistochemistry staining is examined and used as a validated predictive factor. According to clinical data, this marker does not have a high prediction sensitivity or successful translation into the effectiveness of the therapy [9,10,11,12,13,14]. Research searching for clinically appliable predictor factors is highly needed. One significant problem is the high frequency of adverse effects in the III and IV degree of severity through patients treated by ICI [15,16,17]. More specific analysis of cancer–microenvironment and immunity–drug interactions may give knowledge on how to improve clinical benefits from treatment. 

As mentioned above, lung cancer is highly heterogenous. Mutation diversity profile is linked with the presence of driver mutations. *EGFR*-positive tumors include a higher percentage of branch mutations (different from mainly occurred somatic mutations) than trunk mutations (commonly present in all somatic tissue). Circulating tumor DNA (ctDNA) encodes trunk mutations in general and does not reflect tumor heterogeneity [18]. Single-cell transcriptomic analysis of biopsy samples collected from patients with NSCLC in the III/IV disease stage showed significant differentiation in cellular regulation processes, molecular tumor development, and cell phenotype. Several scientific groups instigated studies concerning the changes in dependency quality composition of immune cells in TME according to disease stage [19,20,21]. On the basis of reports about high variability of tumors’ genetic, molecular, and phenotyping profiles amongst patients with the same histopathological cancer type, there is still a necessity to uncover the mechanisms of interaction between tumors and TME’s compartments, improving highly precise basial research with the potential for use in clinical practice [22,23,24]. 

For the analysis of drugs, the most appropriate oncological model is the one that approximates the changes occurring at an advanced stage of the disease. However, in order to learn about new mechanisms of the neoplastic process, all stages of the disease may be considered. Nevertheless, 2D cultures give far less reliable results, even in studies on primary tumors, because they do not retain the native form of intercellular contact. Main strategies for in vitro study on tumor–tumor microenvironment interactions involved the use of tumor spheroids/organoids and patient-derived xenografts. Thorough analysis of pre-clinical models of NSCLC were already published [25,26]. Here, we analyze the main challenges in the field of lung cancer research, taking into account the key features of tumor–microenvironment interactions and technical possibilities for providing in vitro 3D tumor microenvironments for lung cancer cultures. 

## 2. Lung Cancer and Its Tumor Microenvironment

### 2.1. Lung Cancer Origin

**Figure 1:**
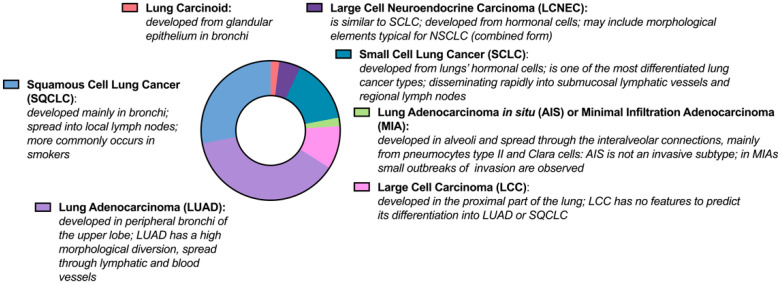
Histological types of lung cancer: NSCLC: SQCLC–28%, LUAD–38%, AIS–2%, LCC–10%; SCLC–15%, LCNEC–5%, and Lung Carcinoid–2% of all cases (this graph was created in GraphPad Prism 9).

Lung cancer is classified in two main subgroups: non-small cell lung cancer (NSCLC) and small-cell lung cancer (SCLC). Every histopathological subtype of lung cancer has a specific localization and different metastatic properties. The representative characteristic of lung cancer subtypes is presented in Figure 1. About 40% of all cases is classified as lung adenocarcinoma. For this histology type, more common genetic alternations include *EGFR*, *BRAF*, *ROS1*, *RET*, *PRKCB*, *NTRK*, *MET*, *HER2*, and *ALK*, as well as somatic mutations in *KEAP1* and *STK11*. For squamous cell lung cancer (SQCLC), mutations generally occur in *PRKCA*, *PKN1*, *FGF*, *FGFR1*, *FGF3*, *MYC*, *CDKN2A*, *RB1*, *TP53*, *CDKN2A*, *NOTCH1*, *MLL2*, and *NFE2L2* genes [6,27]. High tumor mutation burden (TMB) occurs in smokers, which is predicted by long-time exposure on different tumorigenic substances from cigarettes. Clinical sample analysis showed differences not only in somatic mutations type and level, but also in epigenetic regulation, mRNA and miRNA expression, and protein level.

Kim et al. used single cell sequencing of patient-derived material to study genes expression dedifferentiation in different tumor associated cell types based on tumor localization and disease stage. They observed inequal intratumor gene progression levels in accordance with tumor progression. They also analyzed different subtypes of epithelial cells, stromal, and immune cells and matched single-cell phenotypes with the metastatic process. Based on the collected data, Kim et al. proposed twelve fibroblast subtypes, three subtypes of alveolar macrophages, and six dendritic cells (DCs) subpopulation in patient-derived probes in accordance to cell genetic profile [19]. Another single cell-sequencing analysis of lung adenocarcinoma samples defined two main types of intratumor cells: the first similar to epithelial alveolar cells, the second with upregulation of EGFR and JAK/STAT pathways and increased stimulation of the EMT process by transforming growth factor β (TGFβ) and hypoxia-corelated factors [19,20,28]. The key pathways in the epithelial–mesenchymal transformation process involved TGFβ, Wnt, β-catenin, and VEGFR. The main pathways connected via interaction between tumor and TME concerns are p53 protein, Myc, Hippo, and Flower [29]. NSCLC is defined by higher tumor mutation burden than SCLC. SCLC is a neuroendocrine lung cancer subtype quickly metastasized. Low grade neuroendocrine tumor cells are more adhesive than those with high grade, but both subtypes are more likely to generate three dimensional structures. SCLC is less heterogenic than NSCLC [30,31,32]. Herein, in accordance to the presence of major transcriptional factors, five phenotype subtypes of SCLC were investigated: SCLC-A (ASCL1), SCLC-N (NEUROD1), SCLC-P (POU2F3), SCLC-Y (YAP1), and SCLC-I (ASCL1-/NEUROD1-/POU2F3-) [33]. Transcriptomic analysis of patient-derived samples defined combined PLCG-2- positive phenotype, corelated with higher disease aggressiveness in patients with SCLC [34]. Apart from phenotypically differentiated neoplastic cells, there are also cancer stem cells (CSCs). The origin of cancer stem cells depends on the tumor localization in the lungs. In the SQCLC subtype, cancer stem cells are derived from surface epithelium stem cells or submucosal gland. In the SCLC subtype, CSCs develop from Clara cells and pulmonary neuroendocrine cells, and in lung adenocarcinoma from pulmonary stem cell or pneumocytes comprehensively. Lung cancer stem cells typically express EpCAM, CD44, CD90, and CXCR-4. CD44-positive and CXCR4-positive CSCs have the ability to form spatial structures in vitro and in xenograft mice models [35]. CSCs are involved in silencing antitumor immune responses through the production of immunosuppressive and pro-inflammatory cytokines, neovascularization stimulation, and tumor–stromal interactions by TGFβ production. In lung adenocarcinoma, CSCs are involved in immune therapy resistance. Gene profile for LUAD CSCs are better known than for SCLC [36]. CSCs’ induced treatment resistance involves *ALDH1*, *CD44*, and *PTEN* genes, regulated by testis-specific Y-like protein 5 in NSCLC subtypes [37]. CSCs isolated from primary lung cancer samples indicate a correlation between SOX2-positive and NANOG-positive lung adenocarcinomas with high levels of ALDH. In SCLC samples, this correlation was adverse. Tumor cells with high ALDH level formed permanent tumor spheres, while SCLC cells with low expression of ALDH were not able to form spatial culture [38]. CSCs are crucial for tumor progression and drug resistance, but there is still a need for precise methods of obtaining and evaluating their role in cancer diagnostics [35,39,40,41,42,43,44]. 

### 2.2. Tumor Stroma

Fast fibrosis and neovascularization process conditioning in the metastasis formation are associated with higher tumor aggressiveness. Tumor cells are responsible for recruiting stromal cells (myofibroblasts) and immune cells to initiate multi-stage progression. Fibroblasts are involved in extracellular matrix formation due to the synthesis of structural proteins, e.g., integrins, collagen, or elastin [45,46]. Cho et al. showed the myofibroblast impact on fibronectin remodeling through paracrine communication with tumors [47]., A fibroblast subtype crucial for tumor progression include cancer associated fibroblasts (CAFs) developed from myofibroblasts. CAFs are involved in tumor extracellular matrix destabilization and the promotion of angiogenesis. Hu et al. defined three functional subtypes of NSCLC-derived CAFs based on the different expression levels, HGF, FGF7, and p-SMAD2. There was dependency between CAFs functional subtype, targeted therapy effectiveness (all patients had driver mutations), and patients’ pre-treatment condition. CAFs functional differentiation is regulated among others by TGF-β [48,49]. Hao et al. indicated two subtypes of CAFs corelated with NSCLC in accordance to desmoplastic (low- and high-desmoplastic CAFs). These subtypes showed different regulation in tumor progression and matrix destabilization [50]. Sato et al. showed correlation between TGFβ secretion from CAFs and tumor heterogeneity in lung adenocarcinomas. TGFβ stimulated lung adenocarcinomas tumor plasticity into acinar type [51]. Hence, TGFβ mediates a certain role in cell-to-cell cross talk between tumor and tumor microenvironments. It is also involved in extracellular matrix changes through progression and initiate bone-metastasis [52]. CAFs’ co-culture use is required to induce tumor plasticity in 3D models [53,54]. Pericytes interact with TME elements through the production of chemokines and cytokines, but may also polarize into CAFs within stimulation of tumor fibrosis [55]. Pericytes take an important role in tumor progression through the formation of tumor stroma, immune cells regulation, tumor angiogenesis, and neovascularization development and engagement into defeating the brain–blood barrier through lung cancer brain metastasis formation. Bichsel et al. excluded lineage-EpCAM-CD73^+^CD90^+^ perivascular-like cells from patients’ primary lung cancer samples, with high expression of PD-L1, IL-6, and basal α-SMA molecule. Pericytes from patients were proliferated from mesenchymal stem cells, where mice-model pericytes were proliferated from epithelium cells [56]. Under the influence of VEGF, epithelial hyperplasia occurs, the integrity of the blood vessel wall is violated, and a new network of vessels associated with the tumor develops. Tumor vascularization is necessary to maintain the increased metabolism of the tumor by faster delivery of nutrients to the cells. Expansion of blood vessels by the tumor enables metastatic niche formation by tumor-related elements released into the bloodstream.

Furthermore, another extremely important stromal cell subpopulation involved in tumor progression regulation are mesenchymal stem cells (MSCs). During the EMT process, the percentage of MSC subpopulation in tumor microenvironment increased. MSCs produce vimentin, N-cadherin, fibronectin, matrix metalloproteinases, integrins, and smooth muscle actin, which form a foundation of the tumor extracellular matrix [57,58]. MSCs also expressed chemokines involved in the enrollment of immune cells and stromal cells towards increasing tumor cell mobility and overcoming the venous barrier during neovascularization across EMT. Exosomes excluded from MSC are involved in second tumor niche formation and the development of chemoresistance. Exosomes include a shortened cancer repertoire with crucial for disease progression molecules, e.g., proteins and non-coding regulatory RNAs fraction [59]. MSCs, circulating tumor cells, and exosome might be one of the aims for targeted therapy in metastatic lung cancer [60]. 

### 2.3. Immunology of Lung Cancer Tumor

There are two main immune types of cancer: cold tumor and hot tumor. Lack of tumor-infiltrated immune cells, within the absence of pro-inflammatory cytokines and chemokines in TME and expression of immunosurveillance factors, are typical for cold tumors. In contrast, hot tumors are infiltrated by immunosuppressive cells and cytokines, which activate anergy of T cells and make tumors invisible to the host immune system. In NSCLC, the “hot type” is associated with high levels of regulatory T cells in TME within high expression of negative control immune checkpoint inhibitors, PD-L1 and TIM-3, and is more common for lung adenocarcinomas [61]. There is no correlation between immune type of tumor and the appearance of driver mutations (*KRAS* and *EGFR*) [62,63]. Nevertheless, high tumor mutational burden, which is typical for smokers, is connected with better “visibility” of tumors for immune cells [64]. Tumor-infiltrated immune cells may include cancer-infiltrated cytotoxic T cells (CTLs), memory T cells (CD^45^RO^+^), regulatory T cells (CD4^+^CD25^+^), tumor-infiltrated macrophages type M1 and/or M2, and dendritic cells. The infiltration location may appear in the tumor core or its marginal side. The recruitment of immune cells, and their localization by the tumor side, are regulated by immunosuppressive molecules from cancer cells (prostaglandin, histamine, epinephrin, indoleamine 2,3-dioxygenase, arginase, TNF-α, TGF-β, and IL-10) under metabolomic conditions in TME (hypoxia). Immune cells present in TME play certain role in tumor–tumor microenvironment interaction. Every type of tumor-infiltrated immune cells may promote tumor progression or provide antitumor activity. During disease development and progression, cancer cells’ phenotypes become less recognizable for antigen-presenting cells. Involvement of stromal cells (MSCs and CAFs) into interaction with tumor and immune cells translates into the silencing of antitumor immunity. Matricellular proteins, such as collagen type I and III, within CAFs and regulatory T cells form a tumor barrier, which prevents TME infiltration and metastases propagation. Negative correlation between extensive tumor invasion and collagen type V/CAFs tumor barrier, formed by flatting CAFs formation and low concentration of collagen V, was also reported. There were also two types of immune cellular barrier identified, which correlated with the type of matricellular tumor barrier. Collagen I/III-CAFs barrier was correlated with a higher presence of CD3^+^ and CD8^+^ T cells within high expression of PD-L1 and CTLA-4, whereas collagen V-CAFs barrier was associated with regulatory T cell presence and exhausted CD8^+^ T cell within production of immunosuppressives factors [65]. The immune synapse formation between antigen presenting cells (APCs) and lymphocyte is a key step for anti-tumor immune response activation. Tumors use immune checkpoint inhibitory pathways, mediated by cytotoxic T-lymphocyte-associated protein 4 (CTLA-4), PD-1 (programmed death 1), inducible T-cell costimulatory (ICOS), lymphocyte-activation gene 3 (LAG-3), T-cell immunoglobulin, and mucin-domain containing-3 (TIM-3), to prevent T lymphocyte proliferation and activation of natural killer (NK) cells, which are responsible for cancer cell lysis. Tumor immunology is a complex issue, with an unquestionable role in the development of cancer treatment. Immune checkpoint inhibitory (PD-1/PD-L1 and/or CTLA-4 blockade) therapy of NSCLC provides better opportunity for patient response to treatment. Nevertheless, the distinction between immune therapy and chemo/radiotherapy in patients with NSCLC and frequent high severity adverse effect during therapy indicates the need to better understand molecular regulations of ICIs in lung cancer. Here, a critical role belongs to pre-clinical study models. In the presented paper, we describe in brief the main immune cell types and their function in the tumor–microenvironment interaction. Dendritic cells have the highest possibility for antigen presentation to helper T cells. DCs take part in the initiation of adoptive and innate immune response, while the restoration of the adoptive immune response is beneficial for cancer treatment. Functionally mature dendritic cells’ interaction with NK cells provides their activation which contributes in tumor cell lysis [66,67,68]. An important role in the development of immune suppression is held by TME myeloid-derived stem cells (MDSCs), recruited from bone marrow by IL-1 and IL-6, which are the precursor cells for granulocytes, macrophages, and dendritic cells. TME MDSCs convert amino acids into NO and H_2_O_2_, which translates into the inhibition of effector T cells and stimulates the development of highly hypoxic condition. Myeloid-derived stem cells’ phenotype correlates with disease stage. MDSCs are also involved in the inhibition of T cell proliferation, exosome recruiting, tumor metastasis, and neovascularization stimulation through cytokine production under hypoxia condition. MDSCs also stimulate higher expression of PD-L1 on non-specific response’ cells (DCs or macrophages), hence it is a desirable target for novel immune therapeutics [68,69,70,71,72]. Main subtypes of T lymphocyte corelated with lung cancer include Th1, regulatory T cell (T_reg_), and cytotoxic CD8-positive T cells. Th1 subpopulation produce IFN-γ and TNF cytokines, while T_reg_ lymphocyte is responsible for producing interleukin 10 (IL-10) and TGF-β. These cytokines are involved in immune suppression and EMT regulation. B cells are common for lung cancer TME, but there are still not enough data for subpopulation differentiation and its role in cancer immunity [73]. Tumor associated macrophages (TAMs) play a key role in tumor surveillance from immunological control and EMT regulation. There are two main subtypes of infiltrated TME: pro-inflammatory M1- subtype and pro-tumorigenic M2-subtype. Under stimulation by stromal CCL2, the second TAMs subtype produces CCL3, which activates mesenchymal stem cells to produce exosomes involved in the enhancement of EMT [74]. Interaction between tumor cells, CAFs, and TAMs translates into higher levels of VEGF, matrix metal-proteinases (MMP, mainly MMP-3 and MMP-10), and selected chemokines involved in EMT [75]. Another important subject connected with tumor metabolism and tumor microenvironment interaction is hypoxia. Lung cancer is a highly hypoxic cancer type with two-times lower oxygen percentage in the tumor side in comparison to normal tissue. Hypoxia limits immune cell infiltration and conducts tumor surveillance [76]. It is also involved in tumor EMT stimulation and treatment-resistance, mainly in radiotherapy [77]. Cuccarese et al. reported TAM infiltration differences based on cancer molecular type and hypoxia level [78]. Hypoxia-inducible factor 1α (HIF-1α), but also HIF-2α, angiopoietin-2 (Ang-2), fibroblast growth factor (FGF), and insulin-like growth factors (IGF) are involved in cancer progression (vimentin mediated EMT stimulation) and angiogenesis stimulation (VEGF upregulation) under hypoxia condition [79]. Hypoxia stimulates epithelial-mesenchymal polarization in lung cancer, promoting its higher aggressiveness and treatment resistance. High levels of TAMs in TME impacts on the sustaining of tumor hypoxia and exhaustion of TILs. On a metabolomic level, anaerobic tumor cells complement aerobic TAMs. Association between presence of driver mutations and hypoxia levels in tumor cells within production of VEGF differs based on mutational type and localization. A link between factors related to hypoxia and circulating tumor DNA or miRNA was confirmed and can be potentially applied in clinical practice [79]. HIF-1α stimulates tumor neovascularization through involvement of VEGF family, within upregulation of matrix metalloproteinases secretion and higher activity of ERK1/2 pathway [80]. Inhibition of neovascularization and tumor angiogenesis is one of the important clinical strategies in treating advanced lung cancer. VEGFR inhibitors, such as ramucirumab, sunitinib, sorafenib, axitinib, pazopanib, and vandetanib are used in clinical practice [81].

**Figure 2:**
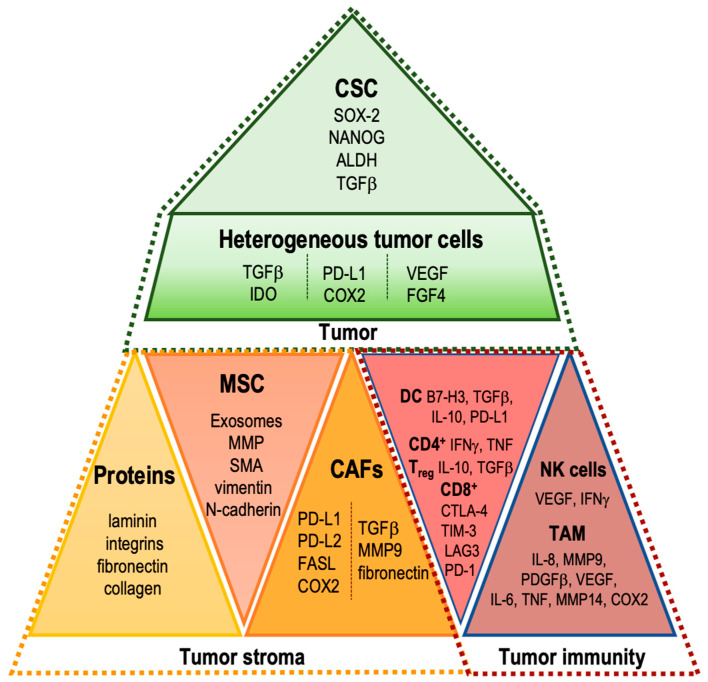
Characteristics of main elements of the tumor microenvironment, involved in tumor progression, angiogenesis, and metastatic promotion.

Briefly, characteristics of the main elements of the tumor microenvironment, involved in tumor progression, angiogenesis, and metastatic promotion, are presented in Figure 2.

## 3. Three-Dimensional Preclinical Models for Lung Cancer Study

**Figure 3:**
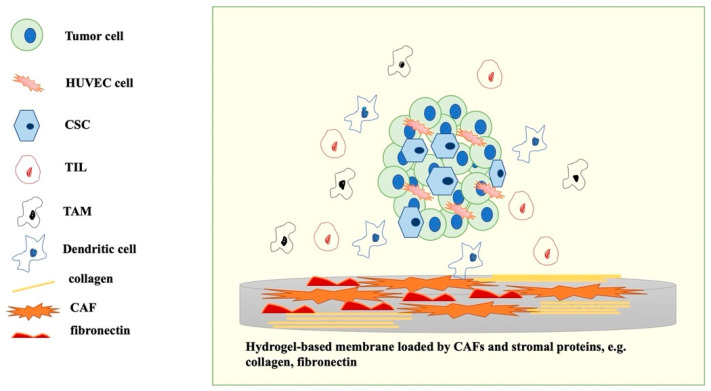
Graphic description of the key elements to mimic tumor microenvironment in vitro.

Herein, mimicking tumor–tumor microenvironment interactions in an in vitro model required the use of cancer cells and tumor stroma such as scaffolds. This can be supplemented by a co-culture of cancer stem cells, immune cells, and normal epithelial cells or normal fibroblasts. Cancer cells can be obtained from commercial cell lines or derived from patients. Almost all cell lines were obtained in 70–80-ss, and the dominant histological type is primary or metastatic lung adenocarcinoma. The use of cell lines limited experiments from molecular dedifferentiation in comparison to clinical practice. Patient-derived cancer cells can be obtained from surgical excerpts, biopsies, or peripheral blood. Schematic abbreviation of significant elements in a 3D pre-clinical model is presented in Figure 3. The type of scaffold and co-cultures used depend on the aim of the study and technical capabilities. New techniques are used for three-dimensional cell culture studies such as spheroids, organoids, patient-derived xenograft models, and in vitro cancer tissue model harvested in bioreactor [82,83,84]. At this point, it should be mentioned that nowadays term “spheroid” is used for 3D cultures derived from commercial cancer cell lines, while “organoid” is used for patient-derived cancer cells. Based on spheroids and organoids, cancer-on-chip and 3D printed tumor constructs were developed [85]. In brief, for 3D tumor–microenvironment interaction study, the use of extracellular matrix and immune cell-co-culture, beside tumor cells, is essential. The type of used scaffold for 3D cell culturing affects the metabolomic of the entire system: the way tumor cells form spatial structures, the penetration rate, and the distribution of medium components. For extracellular matrix imitation, low attachment cell plates, Matrigel, or different modifications of hydrogels containing tumor stromal proteins are more commonly used [86]. Novel methods for in vitro tumor culturing, such as bionic structure, are under investigation. The use of complex tumor microenvironment 3D models require the development of higher-precision analytical technical tools and equipment. Several techniques are used for optical detection of tumor microenvironment metabolic complex, e.g., confocal microscopy, Raman imaging, RPPA, NMR-based imaging, MSI, and MALDI-based imaging [87]. Another method was reported, namely transparent tissue tomography–3D scanning for analysis of immune checkpoint inhibitors monoclonal antibody penetration inside the ex vivo tumor model and permeability of the tumor microvascular system. Ex vivo tumor samples were generated from patient-derived xenograft NSCLC mice [88]. Chen et al. used A549 spheroids to investigate therapeutical effect of hydroxychloroquine on cellular lipidomic. Their matrix-assisted laser desorption/ionization-mass spectrometry imaging showed differences in type and level of lipids according to the spheroids’ region location [89]. A color-coded tumor tissue model for lung adenocarcinoma multicellular spheroids was reported by Chan et al. This method is based on the application of fluorescent proteins, which provides the possibility to monitor interactions between tumors and TME elements within cellular phenotype detection under confocal microscopy [41,90,91]. An important point in tumor–microenvironment interaction studies is metabolite production and distribution between tumor cells and morphological compartments in tumor microenvironments. Tumor spheroids or organoids are typically harvested after 6–7 days, which is enough for metabolites production studies. It should be remembered that not every change at the genetic level is reflected at the protein one. Regarding cell heterogeneity in tumor microenvironment 3D models, the use of tools enabling the simultaneous transcriptomic analysis of several cell types is desirable. Single cell sequencing offers a possibility to identify tumor heterogeneity and TME cellular elements, e.g., creating TME infiltrated immunological cells map [92]. 

The appropriate selection of individual elements, and at the same time, the complexity of the culturing system closely correlates with the set goals of the study. In a 3D tumor microenvironment mimicking model, cancer cells, tumor stromal elements, and tumor infiltrated immune cells may be used, as well as normal epi- or endothelial cells, normal fibroblasts, or cancer stem cells. Nevertheless, the type of cells used in co-culture is predicted by posed hypotheses and research questions. More basial tumor-3D models may be sufficient for cognitive study, but the use of complex organoid or tumor-on-chip models is required for preclinical studies. Well-planned and -conducted basic research translates into a reduction in clinical trials, fewer extra costs, and faster introduction of the drug into clinical practice. The type and mechanical properties of the scaffold used within tumor stromal elements, along with the type of cancer cells used and their co-cultures in spheroids under consideration, significantly impact experiment quality and applicability. 

### 3.1. The Origin of Used Tumor Cells

**Figure 4:**
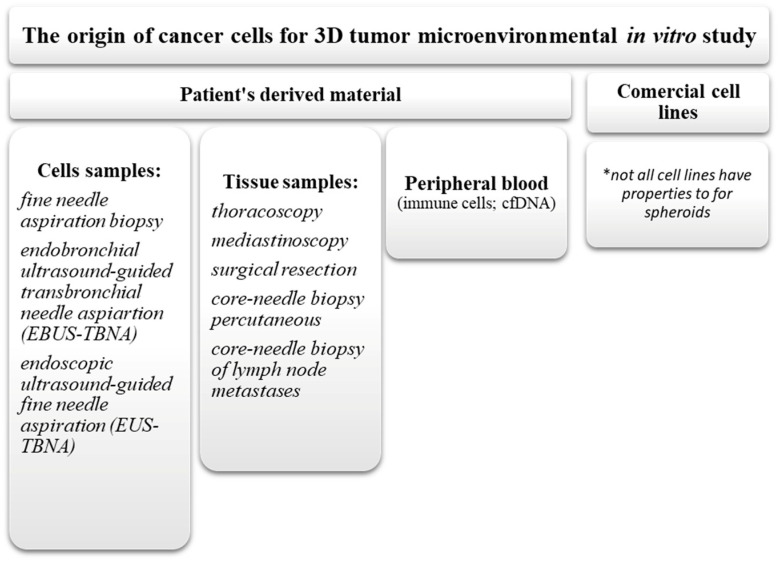
The origin of cancer cell obtained for 3D lung cancer in vitro model formation.

The origin of tumor cells commonly includes commercial cell lines or autologous cells derivate from patient samples collected during surgery (primary tumor model) or from liquid biopsy (often in metastatic disease stage) (Figure 4). Another practice for obtaining ex vivo tumor cells is the use of mouse xenografts. Patients’ peripheral blood (liquid biopsy) is predominantly used for obtaining the mononuclear cells and, less often, for CSCs and exosomes isolation. Liquid biopsy samples also include cancer free DNA, miRNAs, and tumor-related proteins. This method offers the possibility of more personalized studies of cancer genetics, but there is a disadvantage in the standardization of this method between patients [27]. Most of the commercial cell lines were obtained in the 1980s, therefore their genetical profile might deviate from those which occur in patients nowadays. However, the use of commercial cell lines gives a “constant factor” with a characterized genetical and molecular cellular profile, while patient-derived tumor cells represent a personalized preclinical model. As already mentioned, study on advanced stage lung cancer models is critical for application to clinical practice. Herein, cancer cells can be obtained from patient biopsies and collected for diagnostic research. Both the quality of the sample and its qualitative and quantitative content depend on the bronchoscopic method [93]. 

### 3.2. Multicellular Spheroids

The most basic 3D culture model for tumor microenvironmental interaction study consists of multicellular spheroids established on low-attachment well-plates or Matrigel. Low-attachment scaffolds for multicellular spheroid encapsulation can also be used [94,95]. Multicellular spheroids include cancer cells within one or two types of normal cell lines and/or human umbilical vein endothelial cells (HUVECs). One commonly used scaffold mimicking tumor stroma is Matrigel, which includes extracellular matrix proteins (laminin and collagen type IV), proteoglycans, growth factors, etc., whereas CAFs and/or normal fibroblasts can be established on agarose or hydrogels. The most popular “source” of cancer cells in already published research articles are commercial cell lines (e.g., the commonly used lung adenocarcinoma cell line A549) and fibroblast culture and/or HUVECs cells in co-culture [41,54,96,97]. There are some examples of methods for generating multicellular spheroid within more than one co-culture below. Chan et al. proposed a 3D model for testing interactions between cancer cells and TME-elements through the use of four co-culture LUAD cell lines, normal human epithelial cell lines, human fibroblasts, and autologous cancer stem cells in a ratio 1:1:1:0.2. They also proposed hypoxia condition use for cell culturing [41]. Jaromi et al. used three dimensional co-culture aggregate consisting of NHLF, HUVECs, and A549/PC9 in ratio 4:3:3 for ABC transporter analysis in accordance to tumor chemo-resistance [98]. Takahashi et al. reported a center-open disc method for culturing A549-spheroids with high-density fibroblasts co-culture for better analysis of tumor–stromal intercellular communication [99]. Kwak et al. developed a tumor-spheroids model established on fibronectin-based Matrigel within co-cultures of human lung fibroblasts and HUVECs. In this model, cell mobility and structural organization within tumor angiogenesis were preserved [100]. Rebelo et al. developed alginate microencapsulation technic for 3D lung cancer model, established from three types of cells: non-small cell lung cancer cell line, cancer associated fibroblast, and monocytes derived from peripheral blood. In that culture model, monocytes’ polarization into M2 macrophages was observed within the production of cytokines and metalloproteinases connected with EMT [95]. Cho et al. presented the method for preparing an extracellular matrix through preincubation of a tumor cell line with a pre-adipocytes cell line. Next, it was decellularized and used for in vitro culturing with tumor spheroids generated from NSCLC’s cell lines [47,101]. 

### 3.3. Hydrogel Based Technics

Several studies point out the impact of hydrogel scaffold composition on the interaction between a tumor and its stromal elements displayed by different level of cytokines and chemokines production. Herein, hydrogel stiffness and capability to link fibroblast culture are important parameters for 3D culture formation. Park et al. used A549, HUVECs, and human lung fibroblast cell lines to generate spheroids established on hydrogel with vascular-mimicking channels. This study presents a better in vitro model for drug-dose testing [102]. Ferreira et al. developed 3D microspheres from A549, fibroblasts, and bone marrow derived mesenchymal stem cell on hyaluronic microparticles. The proposed microparticles are adequate to use for different co-culture combinations and drug testing [103]. Li et al. created a silk fibroin and chitosan based scaffold forced tumor spheres formation [104]. Dhamec et al. developed an A549 spheroid with human lung fibroblast co-culture in N-isopropylacrylamide-based hydrogel for doxorubicin-drug response analysis. The proposed model allowed scientists to obtain region with hypoxia [105]. Mondrinos et al. proposed a 3D model of lung cancer’s impact on muscle cachexia. For this study, A549 spheroids established on 3D collagen I hydrogel were used. Interaction between co-cultures was studied in a microfabricated multichambered device. The use of a cisplatin-resistance A549 cell line in this model gave a chemoresistance-tumor model in vitro [106]. Temples et al. showed dependency between integrin density in a hydrogel system and NK migration. The type and level of produced cytokines and chemokines related to NK cells differed between each lung cancer cell line used [107]. In recent years, optimalisation of hydrogels for cancer study has been growing. The main points of interest refer to hydrogels optimalisation for better analysis of metabolomic changes, e.g., hypoxia, stress relaxation, and hypothermia, but also as a scaffold for ex vivo xenograft. Selecting the right composition translates into the mechanical properties of the hydrogel and its impact on formation of 3D cancer model. Blache et al. used polyethylene-glycol hydrogels to form a 3D platform for simultaneous analysis of transcriptome and secretome [108]. Hydrogels also became a tool for tumor neovascularization study through the mimicking of blood vessel formation in vitro [104,109,110,111,112,113,114,115,116,117,118]. Development of the liquid phase in hydrogels and tumor-on-chip models provided the possibility for the replacement of the culture fluid and thus ensuring the flow of cellular metabolites and regulatory molecules [88,116,119]. Bioprinting technology gives a compatible platform to create tumor-on-chip, which includes tumor stromal elements and is suitable for obtaining multicellular tumor-spheroids on its surface [120]. Veith et al. developed a spatiotemporal apoptosis mapper based on the tumor-on-chip model for monitoring tumor cell death under chemotherapy or induced by TILs [121]. To imitate metastasis, Ramamoorthy et al. developed a metastatic tumor-on-dish model, which offers the potential to analyze cell phenotypes within DNA pharmacogenetics sensitivity [122]. The possibility of preparing several cultures generated from patients’ samples based on tumor-on-chip methodology was also reported. This fact commands the clinical potential of the tumor-on-chip model in personalized medicine, through next-generation sequencing and a possibility of the precise selection of therapy (targeted or immunotherapy, mainly by ICIs). The use of the tumor-on-chip model involves the need to select a suitable analytical model, which could be performed on chip or off chip. A reliable description of the tumor-on-chip method with its application in clinical practice was provided by Berzina et al. [123]. Another way to obtain cancer cells able to form 3D structures are ex vivo derivate allografts or xenografts. Further reported are methods for obtaining decellularized scaffolding for 3D tumor models from mice-xenografts. Strattman et al. used 3D spheroids on decellularized tissue matrix in vitro models in combination with in silico Boolean model [124]. Goliwas et al. proposed a method for the development of perfusion bioreactor platform to investigate the role of extracellular vesicles in tumor–TME interaction on a lung cancer model performed from commercial lung cancer cell lines, fibroblasts, TILs, and exosomes [125]. Mishra et al. proposed rat-derivate decellularized lung as a scaffold for a 3D lung cancer model based on A549 lung adenocarcinoma cell line, harvested in bioreactor [126,127]. Patient-derived xenograft models are based on the injection of cancer cells into mice models for in vivo tumor harvesting. This method is expensive and has some limitations (not all morphological type of lung cancer can be obtained by this model, mainly NSCLC subtypes) [128,129]. Padhye et al. proposed the development of a 3D TME mimicking lung cancer model obtained from previously harvested tumors in syngeneic mice. Multicellular aggregates consist of all cell types presented in TME. The use of a murine model provides the opportunity to generate tumors with an activated, metastatic-process-controlled epithelial–mesenchymal transformation molecular mechanism. Next stage in vitro 3D culture study allowed identification of miRNA-200 family role and Src-pathway in regulation of mesenchymal tumor cells involvement in EMT. A laminin rich matrix was used in that study [130]. Bioprinting is a complex issue which becomes one of the main currents in development of preclinical models. The technical aspects and application uses of bioprinting methods were presented in review by Augustine et al. [131]. 

A short description of selected cited works is provided in Table 1. Advantages, disadvantages, and potential applications of presented 3D models are described in Table 2.

## 4. Challenges in Lung Cancer Diagnostic and Treatment

There are two main directions of preclinical study of cancer: cognitive, for better understanding of the disease, and applied, for investigation of novel diagnostics and treating tools for clinical practice. However, cognitive and applied study issues follow directly from clinical challenges in lung cancer diagnostic and treatment. One of the significant problems is selecting the key predictor and prognostic factors for personalized clinical practice in every disease stage. The main cause of unsatisfactory results in survival times, despite the availability of effective therapies, is patients’ diagnoses in advanced disease stages within local and distal metastasis. Therefore, establishing changes in the molecular relationship between tumor and its TME during cancer development, and progression in translating this into the clinical parameters of patients’ conditions, is crucial. 

### 4.1. Radio- and Chemotherapy

Surgery resection and stereotactic radiotherapy are common methods for early stage lung cancer treatment. Chemotherapy (e.g., paclitaxel, doxorubicin, carboplatin, pemetrexed, and gemcitabine) is used for adjuvant therapy after surgery resection/radiotherapy and as first-line therapy in non-resectable, non-targetable patients. A mutual challenge for chemotherapy, targeted therapy, and immunotherapy is overcoming the frequent occurrence of severe side effects during treatment. Chemotherapy is conducted with non-selective damage on healthy tissues, which contributes to worsening the condition of patients. According to clinical trial data, adjuvant chemotherapy results in less adverse effects, and is a more effective response to treatment [132]. There is a need to improve drug dosage in accordance to tissue penetration, overcoming blood barrier and mechanical barrier of tumor side possibilities via drugs [133]. Herein, hydrogel-based 3D tumor–TME cultures can be used. One of the main directions nowadays is developing tumor-specific nanoparticles loaded by chemotherapeutical drugs for precision delivery and limitation of adverse effects [7]. The use of the tumor-on-chip model provides an opportunity to monitor drug penetration through the tumor sphere in real time and for analysis of molecular changes by off-chip methods. Better learning of cancer chemoresistance is highly needed. There is a possibility that the use of patient-derived material for 3D in vitro models could lead to better understanding of genetic and molecular mechanisms underlying therapy-resistance. 

### 4.2. Targeted Therapy

Targeted therapy in a group of patients with driver mutations gives them the chance to extend survival time under treatment. Similar to chemotherapy, there is an important challenge to overcome the development of drug resistance in cancer. In is way, the next generations of molecularly targeted drugs are still being developed. The limitation of side effects’ appearance during treatment in this therapy type is also needed. For example, in a group of *ALK*-positive cases, the highest effectiveness according to clinical data was shown for lorlatinib, but the highest percentage of adverse effects was also observed [134]. Crizotinib resistance in *ALK*-positive metastatic NSCLC patients developed in one year. The, the next generation of *ALK*-inhibitors were approved: ceritinib, alectinib, and brigatinib. Based on the possibility to overcome the brain–blood barrier, alectinib showed higher effectiveness in brain metastasis treatment. Therapeutic strategies to overcome resistance mutations include the concomitant use of CDK, c-MET, or mTOR inhibitors [135]. In *EGFR*-positive NSCLC patients, the selection of used inhibitors is based on mutational profiles. Three generations of EGFR-inhibitors were developed. For example, afatinib and dacomitinib are effective in T790M-EGFR mutated patients, but there is a limitation in high doses due to adverse effects. A phase III randomized clinical trial (NCT02296125) comparing osimertinib to gefitinib/erlotinib in overall survival time was conducted in non-treated patients with confirmed mutation in exon 19 deletion or L858R allele. The median overall survival was longer for patients treated by osimertinib. After 3 years from the beginning of receiving treatment, 28% in osimertinib subgroup and 9% in gefitinib/erlotinib subgroup were still receiving the drugs. However, scientists observed high grade adverse effects during therapy in about 40% of patients in all tested groups [136]. Combination therapy with osimertinib and another third-generation tyrosine kinase inhibitors is under investigation in several early phase (I/II) clinical studies. Herein, a sufficient challenge is higher range of adverse effects during combinate therapy. For example, in osimertinib plus savolitinib tests with patients with mutations in *EGFR* and *MET* genes, overall response rate was 30% and high grade adverse effects occurred in 57% [137,138,139]. Another targeted drug for lung cancer treatment complies neovascularization inhibitors, within multiple receptor inhibitors (e.g., sorafenib), small-molecule tropomyosin receptor kinase inhibitors and serine/threonine inhibitors or CDK inhibitors [135]. To summarize in brief, the main challenges in targeted therapy of lung cancer, apart from frequent occurrence of third- and fourth-grade side effects, is the limited ability of drugs to overcome blood-barrier and brain–blood barrier within its cancerous tissue penetration properties. The use of three-dimensional tumor–tumor microenvironment models containing, e.g., blood vessels may be applicable in drug dose optimization or in examining how to improve pharmacokinetic and pharmacodynamic properties of the drug. Use of the tumor-on-chip model and bioprinting techniques based on patient-derived cells, in combination with novel analytical tools (e.g., single cell sequencing), provides the opportunity to understand the mechanism of drug-resistance development in every regulatory level, even epigenomic [19,20,28]. In the past years, one of the main directions in overcoming drugs’ toxicity, is the development of specific matching nanocarriers loaded by, e.g., doxorubicin or paclitaxel. Herein, 3D tumor–TME in vitro models become a cost-effective opportunity to investigate nanocarriers’ penetration rate and mechanism of action in a heterogeneous tumor and its interaction with TME. An important factor, in the limitation of side effect frequency during therapy, is personalized drug-prediction before treatment introduction. The model, potentially applicable into clinical practice, was proposed by Strattman et al. and is described above [124]. Cancer stem cells in three-dimensional tumoroids play a crucial role in drug-prediction testing and drug-sensitivity/resistance testing through a gel-free technique or microfluidic platform according to different research groups [140,141]. 

### 4.3. Immunotherapy

The basis of immunotherapy is restoring the efficiency of the host’s immune system against cancer. It may involve different steps in inducing an immune response. For the enhancement of cancer antigen presentation and recognition, cancer vaccines were developed. Vaccines were produced based on use of tumor antigens, MAGA-A3, MUC-1, TERT, or several tumor-associated antigens (TAA). Nevertheless, major clinical trials were not continued in phase III. In recent years, the concept of combinational use of therapeutical vaccines with ICIs has developed. Saavedra et al. reported a CIMAvax-EGF vaccine which was sufficiency confirmed in advanced NSCLC clinical trials. The role of the CIMAvax-EGF vaccine is stimulation of EGF-antibodies production [142]. The ongoing clinical trial NCT04298606 is focused on CIMAvax-EGF for the prevention of lung cancer development [143]. Another option for the enhancement of antigen presentation is the transfusion of sensitized in vitro antigen presenting cells, but this method is not sufficient for advanced disease stage treatment. Action on immune check points on T cells and specific regulatory receptors on cancer cells indicate the main direction of lung cancer immunotherapy. The main ICIs used in clinical practice concern the PD-1-PD-L1 pathway and CTLA-4. Herein, comparable frequency of high-grade adverse effects during immunotherapy vs. chemotherapy involved advancement in drug dosage and a therapeutical scheme. Data from clinical trials showed no correlation between lower doses of nivolumab and lower frequency of adverse effects. There is a clear need to investigate the correlation between patients’ immune state and regulatory processes developing in TME. The combination of two ICIs, ipilimumab and nivolumab, showed promising results in elongating survival times. Again, the long-term use of immunotherapy resulted in serious, life-threating adverse effects, which might be provoked by over-stimulation of the immune response [11,15,144]. However, better acknowledgement of the regulatory process between tumor–TME-immune host under treatment is highly needed. Another point is introducing ICOS, LAG-3, and TIM-3 potential into clinical practice [145]. Datar et al. reported no correlation between expression of LAG-3 and PD-1 inhibitory axis within independent correlation between expression of PD-1, TIM-3, and LAG-3 and highly proapoptotic T lymphocyte cell type [146]. 

In the selection of therapy, it should be taken into account whether the tumor tissue has inflammatory features. For noninflammatory types, innate or adoptive immunotherapy, CAR-T, and targeted therapies could be used. On the other hand, for inflammatory types, anti-PD-1/PD-L1 and/or anti-CTLA4, active innate (IDO inhibitors and cytokines), or active adoptive immune therapy could be used. Herein, the investigation of high precision predictive factors for clinical practice use by immunoprofiling is needed [147]. The use of autologous cells in three-dimensional tumor models based on liquid-phase tools and various extracellular-matrix compositions is one potential approach to discovering prognostic and predictive factors for immunotherapy clinical use. Investigation of correlation mechanisms between tumors, immune cells, and tumor stromal elements and their translation into organism level has the potential to overcome immunotherapy gaps in clinical settings [148,149,150]. The potential tool to develop high quality analytical platforms may improve through the use of advanced 3D tumor–TME preclinical models.

## 5. Conclusions

We presented critical points for tumor–tumor microenvironmental interaction within brief characteristics of in vitro strategies for obtaining 3D tumor microenvironmental models. The way to choose better pre-clinical models is predicted by the aim of the planned study and is limited by the availability for patient-derived material, technical background, and funding. In the field of cognitive study on lung cancer, future perspectives in tumor microenvironmental interaction studies may include:

- Investigation of CSCs role in second niche formation;
- Analysis of TILs differentiation mechanism in TME;
- Analysis of cells’ phenotype polarization in accordance to culturing conditions;
- Exploring knowledge on tumor–TME interaction;
- Investigation of new regulatory and tumor developmental mechanisms.
- Future perspectives for applied studies on tumor–TME interaction is predicted by clinical trial data. The main hallmarks of lung cancer diagnostics include:
- Prognostic factors from liquid biopsy identification (e.g., potential use of exosomes, cfDNA, and CSC);
- Identification and validation of elements derived from tumor side.
- The challenges in lung cancer therapeutical strategies concern:
- Improvement in pharmacokinetic and pharmacodynamic drugs’ properties;
- Overcoming drug resistance;
- Discovering combined therapies;
- Discovering new regulatory mechanisms in tumor–TME relationships;
- Reduction in adverse effects frequency and grade;
- Drug dosage optimalisation;
- Higher specificity in drug delivery (e.g., specifically matched nanocarriers for drug delivery).

Advantages in bioprinting technologies and a large variety of analytical methods make it possible to provide highly specific and personalized pre-clinical studies, with faster applicability into clinical use and no necessity to involve costly and less effective animal models.

## Figures and Tables

**Table 1:** Short description of selected cited works is provided.

	Cellular Components of the Model	The Aim of the Model Design	Reference
**Multicellular spheroids model**	Three-dimensional co-culture: lung adenocarcinoma cell lines, normal human epithelial cell line, human fibroblast and autologous CSC in a ratio 1:1:1:0.2	Tumor chemo- and radio-resistance analysis	Chan et al. [41]
	NCI-H460 and A549 cell lines established on pre-prepared decellularized tumor-associated matrix	Role of the fibronectin in tumor extracellular matrix	Cho et al. [47]
	Microencapsulation method for obtaining three-dimensional co-culture: NCI-H157, lung derived CAFs, THP-1/peripheral blood derived monocytes	Prediction of chemo- and immunotherapy response	Rebelo et al. [95]
	Three-dimensional co-culture aggregate: NHLF, HUVECs and A549/PC9 in ratio 4:3:3	ABC transporter analysis Tumor chemo-resistance analysis	Jaromi et al. [98]
	Long-term co-culture of A549 spheroids and fibroblasts	Interaction between tumor and tumor-stromal cells (fibroblasts)	Takahashi et al. [99]
	Three-dimensional co-culture: MDA-MB-231, MSCs, HLFs and HUVECs established on collagen-based Matrigel and fibrin gels, with the presence of bioengineered blood vessel	Tumor angiogenesis and blood vessel invasion study	Kwak et al. [100]
**Hydrogel- based technics**	Spheroidal co-culture: A549, human lung fibroblasts cell line, HUVECs, established on hydrogel with vascular-mimicking channels	Drug-dose testing	Park et al. [102]
	Three-dimensional microspheres: A549, fibroblasts, bone marrow derived MSC established on hyaluronic microparticles	Drug testing Adequate method to use for different co-cultures	Ferreira et al. [103]
	Silk fibroin and chitosan-based scaffold	Tumor spheres formation Neovascularization study	Li et al. [104]
	Three-dimensional co-culture: A549 and human lung fibroblast established on N-isopropylacrylamide-based hydrogel	Doxorubicin-drug response analysis Tumor hypoxia study	Dhamec et al. [105]
	A549 (cisplatin-resistant) spheroids established on collagen type I based hydrogel, cultured in advanced microfabricated multichambered device	Study of lung cancer impacts on muscle cachexia Tumor chemoresistance study Tissue anisotropy examination	Mondrinos et al. [106]
	Three-dimensional cell culture: A549 or H1299 with co-culture of NK-92 cells established on peptide-functionalized poly(ethylene glycol)-based hydrogel	Correlation between integrins density in hydrogel and NK cells mobility	Temples et al. [107]
	MSC activated by secretomes from different metastatic niches, established in hydrogel by encapsulation technic	Simultaneous analysis of transcriptome and secretome Examination of correlation between primer and metastatic tumor	Blache et al. [108]
	Tumor-on-chip model	Monitoring tumor-cell death under chemotherapy or induced by TILs	Veith et al. [121]
	Metastatic tumor-on-dish model	Analysis of DNA phenotype changes during metastasis	Ramamoorthy et al. [122]
	Three-dimensional culture: A549 and HCC827 cultured on See porcine material *In silico* Boolean model	Gefitinib sensitivity study Prediction of targeted therapy response	Strattman et al. [124]
	Three-dimensional cell co-culture: A549/H358 and IMR90 or A549/H358, IMR90 and THP-1 cell lines were mixed with collagen-rich extracellular matrices and basement membrane, next cultured in bioreactor	Study on exosomes role in interaction between tumor and tumor microenvironment Exosomes role in tumor immunology	Goliwas et al. [125]
	A549 cells cultured on decellularized rat-derived lung in bioreactor	Mimicking tumor growth in vitro	Mishra et al. [126,127]
	Multicellular aggregates pre-cultured in syngeneic mice were established on laminin rich matrix	Investigation of miRNAs role in EMT process	Padhye et al. [130]

**Table 2:** Advantages, disadvantages, and potential applications of presented 3D tumor–tumor microenvironment in vitro models.

Type of 3D Model	Advanteges	Disadvanteges	Applicability in Lung Cancer Diagnostic and Treatment
Multicellular spheroids	Cancer cells obtained from commercial cell lines within the known molecular profile. Commercial or autologous cells could be used in coculture. Tumor exhibit heterogeneous features. Tumor has hypoxia polarization. Procedure is less complicated than for organoids. No limitation to access to patient derived tumor samples. Cost effective and less time consuming (3 days).	Genetic profile may be different from that in commercial cell lines, obtained on 80s. A constant molecular profile for each commercial cell line allows for reproducible results but does not reflect the clinical reality.	Prescreening activity of potential drug. Gene regulation and molecular changes analysis with gene editing technique (e.g., CRISPR/Cas9). Study on different molecular pathways. The use of commercial cell lines gives the possibility for better statistical analysis and results’ translation, because of maintaining repeatability of cell lines’ genetic and molecular profile.
Organoids	Established from patient derived tissue samples is a personalized in vitro model. Allows obtaining similar to the clinical "population" under laboratory conditions. Has more heterogenic structure than spheroids derived from commercial cell lines. It could contain other than tumor cells (e.g., CSC).	Cell source is highly depending from the quality of diagnostic technique (bronchoscopy) in patients with non-operable lung cancer. The limitation and a high risk of low-quality sample. Tissue samples obtained during surgery resection, performed in lung cancer patients with I-IIIA disease stage, represent different than advanced disease stage’ molecular profile. Time consuming (≥6 days). Access to patients derived samples is needed.	Precise molecular diagnostic for each patient. Potential application in personalized medicine prediction *in vitro.*
Hydrogel-based technics:	Hydrogels can be used as a scaffold for different spheroidal culture and organoids. Both synthetic and natural hydrogels have different stiffness and capacity level, which transmits into the metabolomic of the whole model.	Synthesis and optimization of hydrogel scaffolds require advanced equipment use and is time consuming.	Analysis of the correlation between different types of tumor-stromal elements, tumor cells, tumor-related immune cells in changeable proportions.
Bioprinting (Tumor-on-chip, tumor-on-dish)	Autologous samples are used for generation of bionic tissue/organ samples. Contains liquid phase. Could contain blood vessel mimicking channel. There is a possibility to take two types of analysis: on-chip (limitation of material loos), and off-chip (e.g., analysis of fluid content).	Require high quality material, including patient derived samples. It’s an expensive technique, which require advanced equipment. Interdisciplinary work.	Advanced drug screening. Study on disease development and progression. The possibility of conducting the examination at the level of the whole organ.
Bioreactors	A natural decellularized scaffold is used (e.g., mouse decellularized lungs). Metabolites’ changes can be monitored in real-time observation. Long-term culturing could be provided.	Specialized equipment is required A complex procedure. Required to obtain potential scaffold from animal model.	Drug metabolism in cancer cell and tumor microenvironments screening. Cancer metabolism study.

## Data Availability

Not applicable.
